# Successful minimally-invasive management of a case of giant prostatic hypertrophy associated with recurrent nephrogenic adenoma of the prostate

**DOI:** 10.1186/1471-2490-13-18

**Published:** 2013-04-08

**Authors:** Robert M Learney, Sachin Malde, Mark Downes, Nitin Shrotri

**Affiliations:** 1 Department of Urology, Kent & Canterbury Hospital, East Kent Hospitals NHS University Foundation Trust, London, UK

## Abstract

**Background:**

Benign Prostatic Hypertrophy (BPH) is said to affect at least a third of men over 60. However, the literature contains fewer than 200 reports of prostates over 200g in mass - Giant Prostatic Hypertrophy (GPH). Nephrogenic adenomas are benign lesions of the urinary tract that are believed to represent the local proliferation of shed renal tubular cells implanting at sites of urothelial injury.

**Case presentation:**

We present the first case in the literature of these two rare pathologies co-existing in the same patient and the successful management and 36-month follow-up of the patient’s symptoms with minimally invasive therapy, including the still-uncommon selective prostatic artery embolisation. We also briefly discuss the role of PAX2 in injured renal tissues and nephrogenic adenomas.

**Conclusions:**

Symptomatic Giant Prostatic Hypertrophy (GPH) can be successfully managed with a combination of serial TURPs, 5 *α*-reductase inhibition and selective prostatic artery embolisation (SPAE).

## Background

Giant prostatic hypertrophy (GPH) is the benign hypertrophy of the prostate gland to a mass exceeding 200g. The condition was was originally described by Blanchot in 1952 [1] and has appeared in the English-language literature fewer than 50 times since, although more cases have been reported in the Asian literature (84 in the Chinese [2] and 33 in the Japanese [3]). GPH commonly presents with obstructive lower urinary tract symptoms and hæmaturia. Owing to the size of the gland, definitive treatment usually requires suprapubic prostatectomy, although a case of successful management via 3 separate transurethral resections has been described [4].

Nephrogenic adenoma is a separate disease entity, first described by Davis in 1949 [5] and most commonly comprising small lesions (<1cm) located within the bladder (80%) [6] and histologically resembling distal renal tubules [7]. Whilst nephrogenic adenomas are benign, they can however mimic prostatic adenocarcinoma when located in the prostatic urethra, unless differentiated by immunostaining [6]. For lesions causing obstruction or hæmaturia, endoscopic management is usually effective with the caveat that they can recur in up to 90% of cases [8].

We present the case of a patient who suffered years of irritative lower urinary tract symptoms (LUTS) and hæmaturia from a 460g prostate infiltrated with nephrogenic adenoma but elected not to undergo suprapubic prostatectomy. He was successfully managed by serial TURPs and selective prostatic artery embolisation (SPAE), and has remained symptom free for more than 36 months since.

We also discuss GPH and nephrogenic adenoma, and review the literature surrounding SPAE for intractable hæmaturia of prostatic origin.

## Case presentation

### Initial presentation

In April of 2001, a 62 year old man with a past history of mild hypertension, hyperthyroidism and vitiligo presented to our department via his General Practitioner (GP) with irritative LUTS of 2–3 × nocturia, daytime frequency (once every 1–2 hours) and a PSA of 4.6 ng/mL.

Physical examination revealed an otherwise healthy patient. Digital rectal examination found the prostate to be smoothly enlarged and symmetrical. Q _*max*_ at this time was 14.4mL/sec. Transrectal biopsy of the gland was entirely benign. In addition to continuing the prazosin 2mg BD prescribed for his hypertension, the patient was given a trial period of tolterodine XL 4mg OD and asked to return for review after 6 months.

At this review, the patient’s PSA had fallen to 3ng/mL and he described symptomatic improvement with tolterodine. He was therefore discharged back to the care of his GP.

Unfortunately, the patient’s irritative symptoms returned despite previous pharmacological success, with 3–4 × nocturia, 2–hourly daytime frequency and dribbling urge incontinence. He also described hesitancy and slow flow. No flow testing was performed at this time, but the patient was offered a cystoscopy under anæsthesia with consent to proceed to a TURP should it prove necessary.

Rigid cystoscopic examination under spinal anæsthesia revealed a large, highly vascular prostate protruding into an otherwise normal bladder. Owing to the difficult combination of the vascularity and the prolonged operation time under spinal anæsthesia, only an estimated 50% of the gland was resected, weighing 35g. The complicated nature of his resection was evidenced by a greater than 2 g/dL fall in his hæmoglobin from its pre-operative level of 12.9 g/dL to 10.6 g/dL. His coagulation studies were normal. Histology revealed benign prostatic hyperplasia (BPH) with evidence of minor, focal chronic inflammation.

Just 3 months after this procedure, the patient returned with the same irritative LUTS of urgency and 2–hourly daytime frequency but now with suprapubic pain on micturition as well as intermittent lightly stained frank hæmaturia. Two mid-stream samples sent by his GP were negative for infection. An urgent flexible cystoscopic examination revealed no post-TURP stricturing, but there was a degree of post-operative bladder reaction and a large part of his large vascular median lobe remained protruding into the bladder. His flow rate had also dipped to a Q _*max*_ of 6.8 mL/sec. He was taken off his tolterodine and commenced on finasteride 5mg OD with the caveat that he may warrant a further TURP to complete the resection should his symptoms persist. At a review appointment 6 weeks later, the patient elected to continue pharmacological management rather than to undergo a further TURP. His flow rate improved over the next 3 months to reach a Q _*max*_ of 11.3 mL/sec.

Fortunately, for nearly the next 3 years on finasteride the patient reported good flow, no frank hæmaturia and minimal irritative lower urinary tract symptoms. His Q _*max*_ reached 14 mL/sec, and his PSA remained around 1.7–1.8 ng/mL.

### Diagnosis of giant prostatic hypertrophy and nephrogenic adenoma of the prostatic urethra

In June of 2005, the patient returned to his GP with a few days of frank, heavily stained hæmaturia. A subsequent trans-abdominal ultrasound in our rapid-access hæmaturia clinic estimated his prostate volume at 215 cm^3^ – a dramatic increase from the estimated volume of 70 cm^3^ at his original TURP. However, there was no evidence of upper tract or outflow obstruction with only a very minimal post-voiding residual (however, no quantification was given in the patient notes at the time). Flexible cystoscopy that day revealed an enlarged, highly vascular intravesical median lobe of the prostate with an oozing necrotic mucosal lesion in the prostatic urethra superficially resembling a transitional cell carcinoma.

Transurethral resection of the mucosal lesion under general anæsthesia obtained 4 mm of papillary tissue and 6 g of prostate chippings to check for deep extension. Therapeutic TURP was not considered at this time given the visual appearance of invasive carcinoma. Indeed, histological examination of the papilliform tissue revealed small tubules lined by mildly atypical cells focally extending into muscle, consistent with an adenocarcinoma of the lamina propria and superficial muscularis. The prostatic chips revealed only benign glandular and stromal prostatic tissue with hyperplasia, but no PIN or invasive malignancy.

However, supplementary immunostaining and reexamination of the suspected adenocarcinoma revealed no mitoses within the tissue and the findings were reinterpreted as those of a benign nephrogenic adenoma of the prostatic urethra.

Whilst only a small amount of prostatic urethral tissue was taken for diagnostic purposes, this effectively prevented any further frank hæmaturia for 12 months. An intermittent period of irritative LUTS were managed by decreasing the patient’s caffeine intake. The patient continued to take finasteride 5 mg OD.

In July of 2006 the patient was referred back to our service with 3 days of frank hæmaturia with clots. By the time we saw him in clinic he had been bleeding intermittently for approximately 3 weeks. Fortunately, his hæmoglobin was within the normal range at 13.4 g/dL, and again his coagulation studies were normal. Upper tract imaging was also normal. Given the patient’s previous history, a further TURP was performed in September 2006 which obtained 60 cm^3^ of prostate chips. Histology revealed mainly BPH with a few foreign body granulomata. There was marked proliferation of tubular structures lined by uniform cuboidal epithelium extending into the prostate chips. The findings were compared to those of the previous resection and the consensus was that this represented further recurrence of the patient’s nephrogenic adenoma. There was no evidence of malignancy.

Again, the patient’s hæmaturia was effectively relieved by this partial resection of the nephrogenic adenoma and he remained well for a further 12 months. We were contacted again by the patient’s General Practitioner in September 2007 when his PSA had risen to 6.1 ng/mL without finasteride from a previous level of 1.9 mg/L on finasteride. The finasteride had been stopped for unknown reasons. The patient also described brief periods of frank hæmaturia over the most recent 2 months period, lasting 3–4 days at a time but without clots. His hæmoglobin remained stable at 13.1 g/dL.

**Figure 1:**
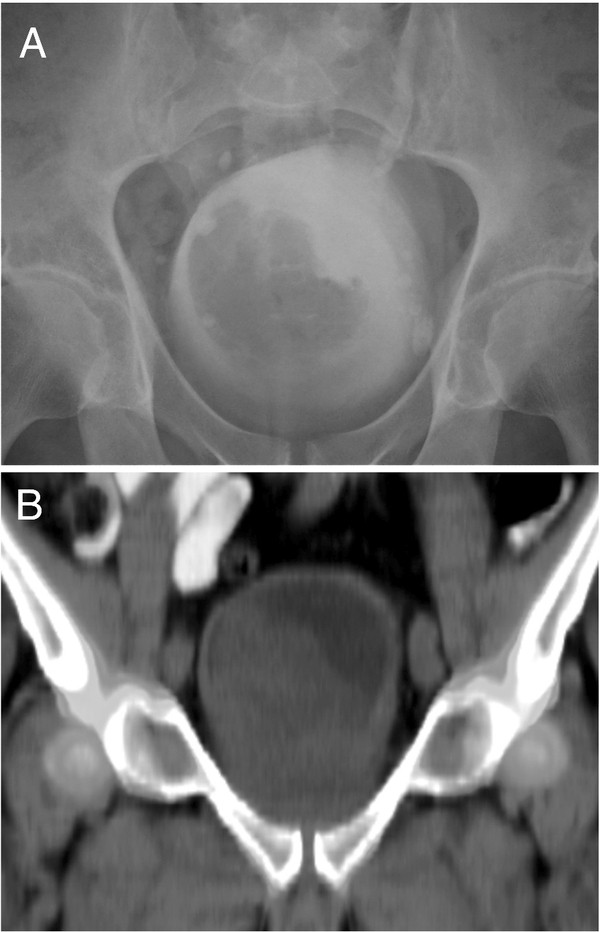
**IVU and CT Images Demonstrating the Intravesical Projection of the Giant Benign Prostate with Nephrogenic Adenoma.** Image **A** - Intravenous Urogram from August 2007. Image **B** - Volumetrically reconstructed CT image from October 2007.

And intravenous urogram and contrast CT of the patient’s abdomen and pelvis revealed a giant hypertrophic prostate, measuring 11 cm × 10.5 cm × 8.5 cm with a massive median lobe protruding into the bladder (Figure 1). The pelvic tissue planes were clearly defined, reinforcing the benign nature of the patient’s pathology. The reconstructed slice-by-slice volume was 460 cm^3^ at this time, double the ultrasound volume of 215 mL measured just 2 years previously.

Most surprisingly, the patient reported only minimal irritative LUTS (1 × nocturia). A further TURP was undertaken in December 2007 to ensure no malignant change. Owing to the vascularity of the recurrent tumour, the patient experienced 4 days of prolonged post-operative hæmaturia requiring irrigation, but without any significant fall in his hæmoglobin (12.3 g/dL).

Once again, the histology was compared against the 2 previous biopsy specimens, and deemed to be consistent with the diagnosis of a recurrent nephrogenic adenoma of the prostate gland.

The patient was recommenced on long-term finasteride, but by early 2009 this was failing to control his increasingly frequent episodes of macroscopic hæmaturia with clots, now at least once every few weeks. The patient was reluctant to undergo open suprapubic enucleation of the giant adenoma, the standard surgical management for this condition, but allowed us to perform a selective prostatic artery embolisation in March of 2009, following preliminary MR angiography.

### Selective prostatic artery embolisation to control hæmaturia

The procedure was performed under local anaesthesia using a unilateral approach via a 5Fr catheter introduced into the right common femoral artery. This gave access to both internal iliac arteries allowing selective bilateral catheterisation of the prostatic vessels with a coaxial microcatheter.

**Figure 2:**
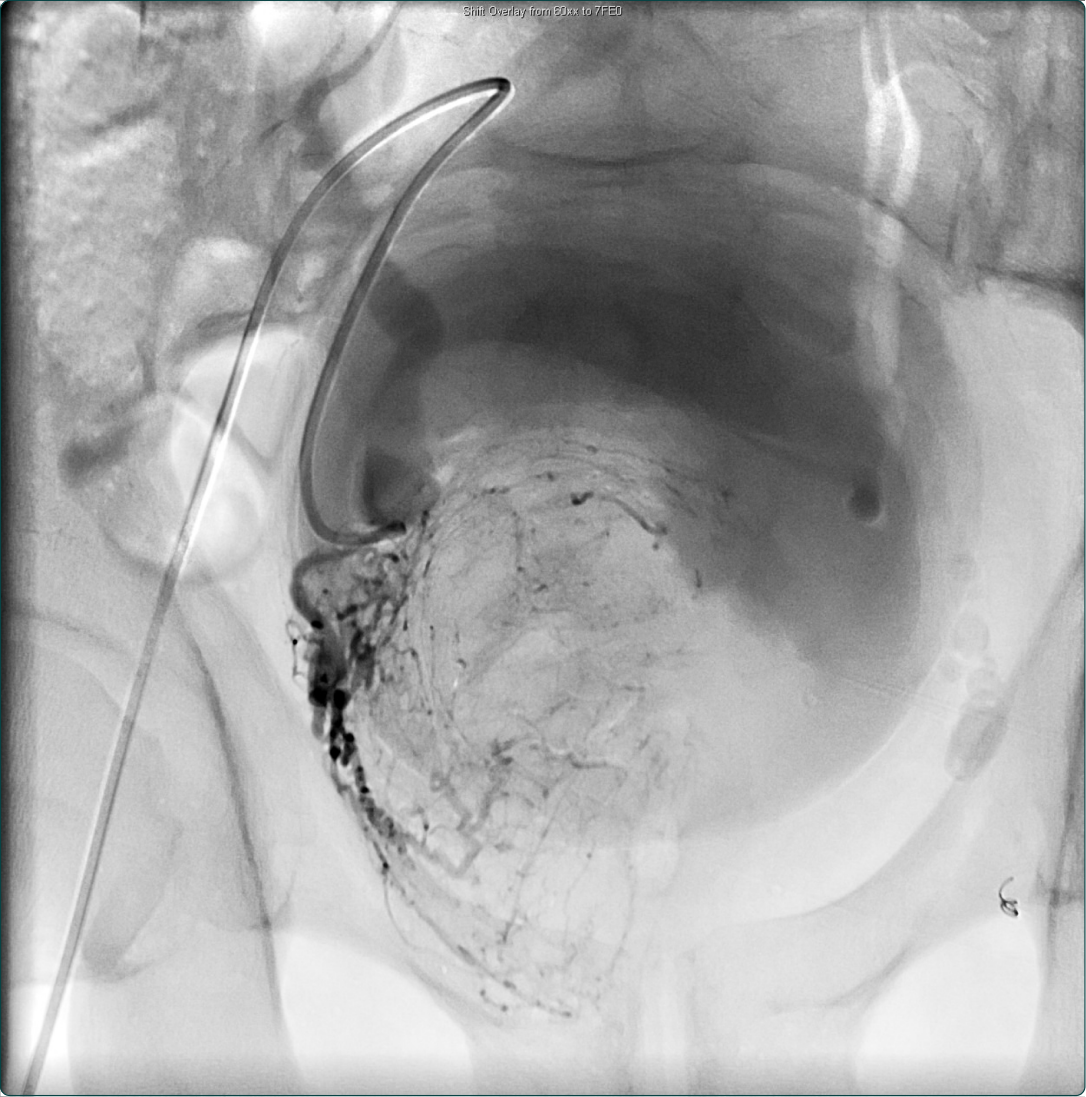
**Selective Prostatic Artery Embolisation.** Angiographic image from the embolisation procedure undertaken in March 2009 demonstrating the dense arborisation of the right prostatic artery supplying the large intravesical body of the giant prostate and nephrogenic adenoma. Image taken immediately prior to microsphere injection.

The left prostatic vessels had a relatively small arborisation which was occluded with 500 *μ*m polyphosphozene-coated hydrogel microspheres (Embozene^®;^, CeloNova BioSciences) and an embolisation coil (VortX^®;^ 18 Vascular Occlusion Coil, Boston Scientific). The right sided supply was more profuse and was believed to supply the majority of the prostatic lesion (Figure 2). This was occluded with 500 *μ*m polyphosphozene-coated hydrogel microspheres and 2 embolisation coils (MReye^®;^, Cook Medical) with a radiologically satisfactory outcome.

The patient was discharged home later that afternoon. An MR examination 2-months post-procedure demonstrated hæmorrhage in the posterior right side of the prostate, the site of the most prominent vasculature, in keeping with a successful procedure. His hæmaturia resolved following embolisation although he described irritative LUTS of dribbling urge incontinence 3–4 ×/week and 2–3 × nocturia in the early post-operative period. These symptoms soon resolved entirely on tolterodine XL 4mg OD.

**Figure 3:**
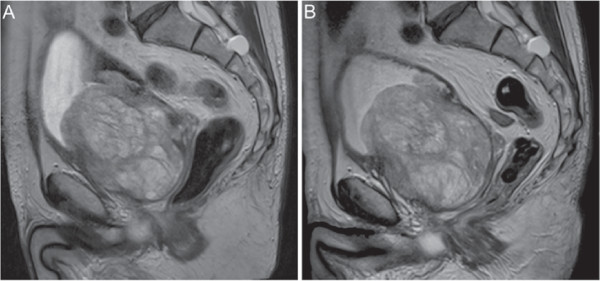
**Sequential Saggital T2 MR Imaging of the Patient’s Giant Prostate.** Image **A** - MRI from September 2008 (6 months prior to embolisation). Image **B** - MRI from April 2011 (25 months after embolisation). Note the minimal change in volume and signal density between the two images.

Serial MR imaging over the intervening 36 months has demonstrated no significant change in the size of his giant prostate, which still measures approximately 460 mL in volume (Figure 3). However, he remains entirely asymptomatic with no further hæmaturia or other LUTS on finasteride 5 mg OD and tolterodine XL 4mg OD. His most recent Q _*max*_ was 12.4mL/sec in January 2010, with a residual volume of 65 mL, and his PSA remains low at 2.4 ng/mL as of January 2013.

## Discussion

Giant prostatic hypertrophy (GPH) is a poorly explored phenomenon, without large case series or definitive histological studies to define this as more than a rare subtype of BPH where the gland exceeds 200 g in mass.

GPH commonly presents with obstructive lower urinary tract symptoms and hæmaturia. Owing to the size of the gland, definitive treatment usually requires suprapubic prostatectomy [2][4], which our patient was reluctant to undergo. As a result, we were required to manage his GPH symptomatically with a combination of pharmacotherapy, repeated de-bulking TURPs for biopsy and therapeutic purposes, and finally selective prostatic artery embolisation for his recurrent hæmaturia, the first such case in the literature.

**Figure 4:**
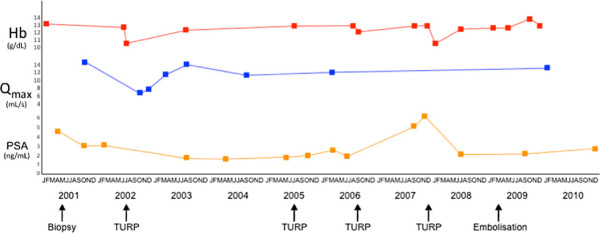
**Patient Timeline.** Graphical representation of the patient’s treatment over 10 years from January 2001 – December 2010. The image provides an overview of the patient’s Hæmoglobin (red), Uroflowmetry (blue) and PSA results (orange) along with the key events in his treatment (Transrectal Biopsy, TURPs and Embolisation). Where multiple Hæmoglobin results were available for a given month, the lowest value was chosen to represent the worst-case scenario.

Whilst we understand that standard operative management of BPH would have had us definitively resect our patient’s prostate at the operation in July 2002, this was limited by a combination of a highly vascular gland, prolonged operating time and the choice to use spinal anæsthesia. Furthermore, prior to the development of *giant* hypertrophy, the patient’s symptoms were predominantly irritative rather than obstructive, as confirmed by the good pre-operative flow result (Q _*max*_ of 14.4mL/sec) (Figure 4). We presented the patient with the opportunity for an early repeat TURP to complete the initial procedure which he declined owing to subjective symptomatic improvement.

By the time the patient required further transurethral intervention, the combination of nephrogenic adenoma and giant hypertrophy further complicated the procedure and ruled out standard management techniques. Of note, even at this stage the patient’s symptoms defied the common presentation for this condition and were predominantly irritative with hæmaturia, rather than obstructive.

### Nephrogenic adenoma

Nephrogenic adenomas are believed to represent shed renal tubular cells implanting into areas of damaged urothelial integrity [7]. Predisposing factors are known to include chronic inflammation, infection, calculi and urological surgery including TURP and renal transplantation [9].

Most features of our patient’s history are typical for recurrent nephrogenic adenoma. He was a middle-aged male with an initial urothelial insult in the form of a transurethral resection of the prostate in July 2002. The interval between this initial transurethral resection and the diagnosis of nephrogenic adenoma was 35 months, and his two subsequent recurrence-free intervals following TURP de-bulking were 14 months each.

Our patient’s PSA has remained between 1.7ng/mL and 2.4 ng/mL whilst on finasteride, with a brief rise to 6.1 ng/mL in late 2007 when this was temporarily halted (Figure 4). This reflects the benign nature of giant prostatic hypertrophy and the low to zero PSA expression in nephrogenic adenomas.

As in our case, macroscopic features consistent with nephrogenic adenoma included papillary and/or ulcerated lesions of the urinary epithelium which may superficially resemble prostatic adenocarcinoma. The microscopic features of resemble remnants of embryonal renal tissue, with small tubules located in the lamina propria and occasional papillary foci or thyroidisation. The lining cells are often cuboidal without atypia or mitoses. Nephrogenic adenomas also commonly express PAX2, a key renal embryonal transcription factor, which has come to be recognised as a specific and sensitive diagnostic marker for these lesions [10].

These histological features were present in our patient, but unfortunately our unit did not stain for PAX2 at the time, which would have served as an interesting but ultimately unnecessary additional piece of information in this case. For a thorough review of the histology of nephrogenic adenoma, see Kunju (2010) [6].

The largest case series of nephrogenic adenomas isolated to the prostate gland itself include 26 patients from The Johns Hopkins Hospital [11], and 8 cases from The University of Texas MD anderson Cancer Center [12]. Whilst the histological features described in these cases match those found in our patient, there have been no prior reports in the literature of nephrogenic adenomas occurring in giant prostatic hypertrophy, nor their treatment by selective prostatic artery embolisation.

### Physiological role of PAX2

The nine PAX (paired homeobox) genes are a series of transcription factors that are critical in orchestrating normal embryogenesis in animal species. Each one appears to be expressed in a limited and specific range of developing tissues, with PAX2 apparently responsible for eye, ear, central nervous system and urogenital tract development [13].

Expression of PAX2 appears to be temporally and spatially modulated in the embryonic kidney by the expression of the Wilms’ Tumour protein (WT-1) [14]. These two factors coordinate to regulate the branching and survival of the ureteric bud and the differentiation of mesenchymal cells to epithelial cells [15] before PAX2 expression is then totally suppressed outside the collecting system. Over-expression of PAX2 beyond embryogenesis only appears to be seen in pathological conditions, including renal cell carcinomas, polycystic kidney disease and Wilms’ tumour.

However, and perhaps of greatest interest in our particular case, *in vitro* studies have demonstrated that PAX2 appears to be normally re-expressed in adult tubular cells following ischæmia/reperfusion injury [13].

This could well explain why a transcription factor whose expression beyond the renal collecting system normally terminates during embryogenesis appears to be re-expressed in nephrogenic adenomas and lead to such characteristic histological features - the shed tubular cells are attempting to regenerate normal tubular tissues at their site of implantation.

Certainly more work needs to be done to explore the specific growth factor environment which allows nephrogenic adenomas to take root and to reoccur despite multiple resections, but our particular case does suggest a possible link between the nesting of nephrogenic adenoma precursor cells in our patient’s TURP-traumatised prostate and the subsequent development of his giant prostatic hypertrophy.

### Management of refractory prostatic hæmaturia

Traditional management options for BPH-related LUTS include pharmacotherapy (*α*-blockade and 5 *α*-reductase inhibition) and transurethral resection. Our patient was managed quite effectively on continuous 5 *α*-reductase blockade (finasteride) and 2 interim de-bulking TURPs between September 2006 and March 2009, after which his hæmaturia became refractory to pharmacotherapy.

Cases of TURP and/or fulguration-refractory gross hæmaturia as experienced by our patient are more difficult to manage, with only limited options available. Intravesical chemo-cautery with alum, formalin or silver nitrate have been described in the literature, along with hydrostatic pressure or balloon tamponade [16]. Depending on the particular therapy selected, adverse effects can include allergic reactions, aluminium toxicity, bladder fibrosis and urethral stricturing caused by the instillation agent, to renal impairment and bladder rupture. However, none of these reports specifically addressed giant prostatic hypertrophy.

### Selective prostatic artery embolisation

The use of selective internal iliac arterial embolisation is in widespread use for the treatment of symptomatic uterine leiomyomata. It has also been described for the management of intractable hæmorrhage secondary to advanced pelvic malignancies, with good long-term outcomes [17].

There are however few studies exclusively reporting on the management of refractory gross hæmorrhage of prostatic origin by selective prostatic artery embolisation (SPAE). The largest case series to date was published in 2008 [18], involving 8 patients in total (6 with post-radiotherapy prostatic adenocarcinoma, 2 with BPH) and ascribing a 100% immediate cessation rate to SPAE following failure of conventional therapies. These benefits extended out to a median of 20 months follow-up in 6 of the 8 patients (1.5–86.3 month range).

The series reported by Liguori *et al*. in 2010 did include 15 patients with prostatic adenocarcinoma, but did not break down their outcomes according to primary pathology. They did achieve an 83% immediate control rate following selective internal iliac artery embolisation across all 44 patients in their study, but at a mean follow-up interval of 10.5 months (1–97 month range), permanent control was only achieved in 19 (43%).

There are other single case reports in the English-language literature specifically describing SPAE for prostatic hæmorrhage refractory to other therapies, with generally high levels of initial success, but listing them all would not be useful here.

SPAE has also been employed for the primary management of BPH without hæmaturia. A 2011 report by Pisco *et al*. described 15 patients with symptomatic BPH treated with SPAE who demonstrated moderate improvements in IPSS and Q _*max*_ that were sustained at a mean of 7.9 months follow-up (3–12 month range). In this study however, there was a clinical failure rate of 28.6% (4 patients) and one major complication of bladder wall ischaemia [19].

Whereas all of these studies are limited by their short follow-up periods, our case is the first to demonstrate the durable long-term results of SPAE in the treatment of refractory hæmaturia and LUTS secondary to GPH and nephrogenic adenoma. Our patient had recurrent rapid prostatic re-growth over the 4 years from 2005–2009, and despite 2 TURPs for debulking and symptom control, his hæmaturia eventually became refractory to pharmacotherapy with 5 *α*-reductase inhibitors. 36 months following embolisation, we have found no further increase in his prostatic volume on serial MRIs (but also no reduction), no further episodes of hæmaturia and no recurrence of his irritative LUTS or decline in his Q _*max*_.

## Conclusions

We have demonstrated that it is possible to manage irritative LUTS and intermittent frank hæmaturia from the giant hypertrophied prostate in the long term with a combination of trans-urethral resections to de-bulk the tumour, 5 *α*-reductase blockade to reduce tumour vascularity and finally selective prostatic artery embolisation to control refractory hæmaturia when open enucleation is not an option.

This case serves to promote the acceptability of selective prostatic artery embolisation as a safe, effective and durable treatment option for managing hæmorrhagic prostatic pathologies, particularly in patients for whom other forms of therapy are not possible.

## Consent

Written informed consent was obtained from the patient for publication of this case report and any accompanying images. A copy of the written consent is available for review by the Series Editor of this journal.

## Competing interests

The authors declare that they have no competing interests.

## Authors’ contributions

RML and SM drafted the report. MD provided expert radiological input. NS reviewed and edited the manuscript. All authors approved the final document.

## Pre-publication history

The pre-publication history for this paper can be accessed here:

http://www.biomedcentral.com/1471-2490/13/18/prepub
